# Unveiling the Current-Phase Relationship of InSb Nanoflag Josephson Junctions Using a NanoSQUID Magnetometer

**DOI:** 10.1021/acs.nanolett.5c03765

**Published:** 2025-09-10

**Authors:** Andrea Chieppa, Gaurav Shukla, Simone Traverso, Giada Bucci, Valentina Zannier, Samuele Fracassi, Niccolo Traverso Ziani, Maura Sassetti, Matteo Carrega, Fabio Beltram, Francesco Giazotto, Lucia Sorba, Stefan Heun

**Affiliations:** † 19004 NEST, Istituto Nanoscienze-CNR and Scuola Normale Superiore, Piazza San Silvestro 12, 56127 Pisa, Italy; ‡ Dipartimento di Fisica, Università di Genova, Via Dodecaneso 33, 16146 Genova, Italy; ¶ CNR-SPIN, Via Dodecaneso 33, 16146 Genova, Italy

**Keywords:** Planar Josephson junctions, Superconducting quantum interference devices, InSb nanoflags, Current-phase relationship, Magnetometers

## Abstract

Planar Josephson junctions (JJs) based on InSb nanoflags have recently emerged as an intriguing platform in superconducting electronics. The knowledge of the current-phase relationship (CPR) of such hybrid junctions is crucial for their applications. This letter presents the fabrication and investigation of superconducting quantum interference devices (SQUIDs) employing InSb nanoflag JJs. The observed features are well reproduced through numerical simulations. By measuring interference patterns in both symmetric and asymmetric SQUID configurations, we extract unprecedented details of the junctions’ CPRs. Our results demonstrate the skewness of the CPR, showing significant contributions from higher harmonics. We explore the magnetic field response of the devices across a wide range of fields (±30 mT). Finally, we assess the flux-to-voltage sensitivity of the SQUIDs to evaluate their performance as magnetometers, identifying a magnetic flux noise of $S_{\Phi }^{1/2}=4.4\times 10^{-6}\Phi_{0}/\surd Hz$ . These results showcase potential applications in nanoscale magnetometry.

## Graphical abstract

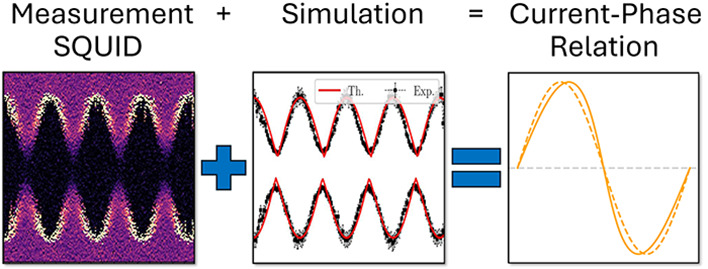

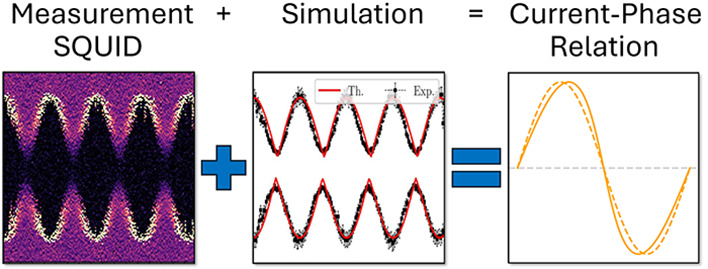

Superconducting quantum interference devices (SQUIDs) are one of the most important classes of devices in quantum technologies. The fundamental component of a SQUID is the Josephson junction, which is created by sandwiching a normal (nonsuperconducting) material between two superconducting electrodes. When two Josephson junctions are connected in parallel to a loop, a dc-SQUID is formed.^1^ This type of device is the most sensitive magnetometer,^2,3^ with crucial applications in scanning probe microscopies^4–6^ and superconducting electronics.^7,8^ Furthermore, SQUIDs in highly asymmetric configurations, where one arm of the SQUID carries a supercurrent significantly larger than the other, have been explored as a way to investigate a fundamental property of Josephson junctions: the current-phase relation (CPR).^9–16^ This key quantity is challenging to test directly in experiments, as recently discussed.^15^ With its small effective mass, narrow bandgap, and significant spin–orbit interaction, InSb has emerged as a highly sought-after material for applications in high-frequency electronics and spintronics.^17^ Additionally, with a large Landè *g* factor, superconducting-semiconducting hybrids incorporating InSb have been proposed as a platform to host topological superconductivity and search for Majorana Fermions,^18–21^ motivating the investigation of this material in conjunction with superconductors. Recently, InSb nanoflags have surfaced as a promising platform featuring quasi-2D electronic transport. These InSb nanoflags, epitaxially grown by chemical beam epitaxy, are free-standing semiconducting nanostructures.^22–26^ There, ballistic Josephson junctions with InSb nanoflags have been fabricated,^27^ and nonreciprocal transport along with half-integer Shapiro steps has been reported.^28,29^ A nonsinusoidal CPR was proposed to elucidate these observations. However, no direct measurement of this CPR has been reported, indicating that experimental studies remain extremely important. Here, we provide this information, on devices that we fabricated following an identical protocol as in previous works.^27–29^

In this letter, we have fabricated and investigated SQUIDs that incorporate Josephson junctions based on InSb nanoflags, in various geometrical configurations, scrutinizing their behavior under the influence of back-gate voltage variations and external magnetic fields. An asymmetry between the two arms can be introduced by considering different geometrical aspect ratios (length/width) of the two Josephson junctions forming the SQUID. By a combined experimental and theoretical approach, inspecting the interference patterns we have extracted unprecedented details on the CPR of these junctions. This approach avoids possible drawbacks^15^ of standard procedure.^10^ Interestingly, the resulting CPRs are highly skewed, showing significant contributions from higher harmonics. Additionally, we explore novel applications of these hybrid structures in detecting minute magnetic signals, thereby evaluating their performance as magnetometers in nanoscale devices.

The SQUIDs investigated are based on Josephson junctions with InSb nanoflags as normal material and niobium (Nb) as superconducting material (measured *T*_*c*_ = 8.1 K). The InSb semiconducting nanostructures possess a zinc blende crystal structure. They are typically 2.8 ± 0.2 μm long, 470 ± 80 nm wide, 105 ± 20 nm thick, and characterized by quasi-2D transport.^22^ Control over the carrier density in InSb nanoflag-based Josephson junctions is obtained by capacitively coupling a p-type doped silicon back-gate through 285 nm of SiO_2_. In the depletion region, when the back-gate voltage *V*_bg_ is below a threshold voltage *V*_th_ (*V*_bg_ < *V*_th_), the conductance of the junction is zero. Increasing the back-gate voltage opens the semiconducting channel, and a steep increase in the conductance is observed.

Because of their elongated shape, nanoflag-based Josephson junctions can be fabricated in different configurations. The “narrow” configuration (JJ1 in Figure 1(b1)), extensively investigated in previous works,^27–30^ is obtained by depositing the superconductor along the shorter direction of the nanoflags. This limits the contact width to ∼ 600 nm, but grants the possibility of tuning the spacing of the Nb-electrodes from micrometers (“Hall bar” or long junction regime) to tens of nanometers (short junction regime). A “wide” configuration (JJ2 in Figure 1(b2)) is obtained by rotating the nanoflags 90 deg, allowing for larger contact widths, up to 3 μm, with an electrode spacing of submicrometer size.

**Figure 1:**
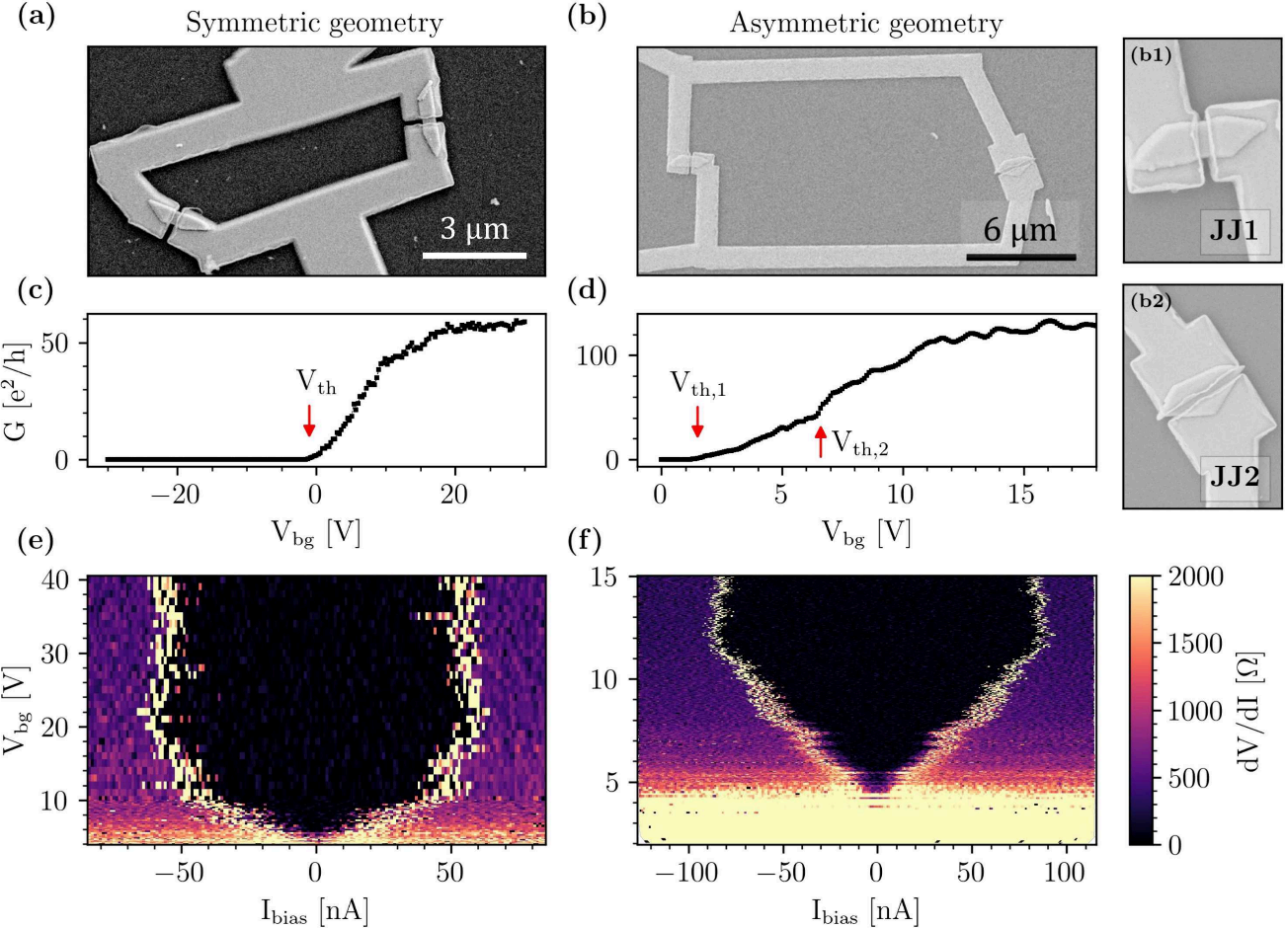
(a), (b) Top-view scanning electron microscopy images of the devices characterized in this work. (a) SQUID in a symmetric geometry. (b) SQUID in an asymmetric geometry. (b1), (b2) Enlarged views of the two Josephson junctions forming the asymmetric SQUID. (b1) Narrow configuration. (b2) Wide configuration. (c), (d) Conductance vs back-gate voltage for (c) the symmetric and (d) the asymmetric geometry, measured at *T* = 2 K. Current bias 100 nA. (e), (f) Color maps of the differential resistance (*dV*/*dI*), for the device in (a) and (b), respectively, as a function of back-gate voltage, obtained as the numerical derivative of the measured *V*–*I* curves. *T* = 350 mK.

This degree of freedom in the configuration is used to realize two SQUID geometries: symmetric and asymmetric. In symmetric geometry, both Josephson junctions have a narrow configuration, as shown in Figure 1(a), with width *W*_1_ = *W*_2_ = 380 nm. The total loop area is *A*_geo_ = 13.6 μm^2^. In the asymmetric geometry, shown in Figure 1(b), instead, one junction has the wide configuration (*W*_1_ = 1.7 μm), while the other the narrow one (*W*_2_ = 530 nm), with area *A*_geo_ = 118 μm^2^. Additional information on fabrication methods, with a table of full geometrical parameters, is available in Section S1 of the Supporting Information (SI). Section S2 of the SI provides details on the measurement methods.

We first characterize the SQUIDs by investigating their transport properties at zero magnetic field. As SQUIDs consist of the electrical parallel of two Josephson junctions, the measured conductance can be expressed as the sum of each junction’s conductance:

1 $$G_{S}\left(V_{\mathrm{th,}1},V_{\mathrm{th,}2}\right)=G_{\mathrm{JJ,}1}\left(V_{\mathrm{th,}1}\right)+G_{\mathrm{JJ,}2}\left(V_{\mathrm{th,}2}\right)$$

where the voltage threshold *V*_th,i_ of each junction has been introduced (see Section S3 of the Supporting Information). In measuring the conductance vs back-gate curves in the normal state for the symmetric geometry, a single threshold is found (red arrow, Figure 1(c)). In contrast, in the asymmetric geometry, two different threshold values are extracted (red arrows, Figure 1(d)). Hence, in the asymmetric SQUID, there is a range of back-gate voltages for which only one arm of the interferometer is conductive. Two distinct voltage thresholds can be attributed to fabrication inhomogeneities that would result in the conduction band edges of the two nanoflags not being perfectly aligned. This, in turn, would cause the chemical potential to be brought into the band gap at two different back-gate voltages for the two junctions.

Looking at superconductive properties, the Josephson effect at zero magnetic field is investigated by measuring voltage–current (*V* – *I*) characteristics as a function of the back-gate voltage in a DC setup. At the temperatures explored here (*T* = 350 mK and above), no significant differences are observed between the switching and retrapping currents, which places these SQUIDs in the nonhysteretic regime. The differential resistance, obtained by numerical differentiation of the measured *V* – *I* curves, is plotted against the voltage of the back-gate and the current bias in Figure 1(e) and Figure 1(f) for the symmetric and asymmetric device, respectively. The back-gate modulates the critical current in both configurations to pinch off, as expected.^27^ Increasing *V*_bg_, the critical current presents a nonmonotonous behavior, reaching a maximum value of 20 and 12 V for the symmetric and asymmetric case, respectively, and then slightly decreases. This modulation allows to exclude Nb accidental shorts or other transport channels different from the InSb nanoflags.

Before discussing the experimental results with an applied perpendicular magnetic field, it is convenient to introduce the key points of the SQUID interference. The total supercurrent flowing through a SQUID, *I*_*S*_, is the sum of the supercurrents carried by each arm, and is a function of the superconducting phase drop across both junctions, φ_*i*_,

2 $$I_{S}\left(\varphi_{1},\varphi_{2}\right)=I_{1}\left(\varphi_{1}\right)+I_{2}\left(\varphi_{2}\right)$$

The superconducting phase drops φ_1_ and φ_2_ are linked via the flux quantization condition

3 $$\varphi_{1}-\varphi_{2}=2\pi \frac{\Phi }{\Phi_{0}}$$

where Φ_0_ = *h*/2*e* ≃ 2.068 mT μm^2^ is the superconducting flux quantum and Φ = Φ_ext_ + *LI*_circ_ the total flux enclosed in the SQUID loop, which is the sum of the externally applied flux Φ_ext_ and an induced flux *LI*_circ_. The induced flux is due to a supercurrent circulating in the loop, *I*_circ_ = (*I*_1_ – *I*_2_)/2, and the total inductance *L*, expressed in terms of a geometrical and a kinetic contribution. The impact of a finite inductance on the phase difference reported by eq 3 is given by the inductance parameter β_L_ = 2*πLI*_c_/Φ_0_. In our case, considering that *L* is of the order of 10pH (Section S4 of the Supporting Information) and *I*_c_ of the order of 100 nA, we get β_L_ ≈ 10^–3^. Thus, corrections to the enclosed magnetic flux due to the self-inductance can be neglected. In the following we will consider Φ ≈ Φ_ext_. The critical current of the SQUID is obtained by maximizing the total supercurrent over the phase difference φ_1_,

4 $$I_{c}\left(\Phi \right)=\max_{\varphi_{1}}I_{S}\left(\varphi_{1},\Phi \right)$$

and is a function of the enclosed flux only. Thus, the shape and the features of the interference pattern $I_{c}\left(\Phi \right)$ depend directly on the CPRs $I_{i}\left(\varphi \right)$ of the two Josephson junctions forming the SQUID loop.

A perpendicular magnetic field is applied to the sample to measure the interference pattern, and the corresponding *V* – *I* curves are measured while the magnetic field is swept. The results for symmetric SQUID are presented in Figure 2, which shows the interference pattern for two values of *V*_bg_. At *V*_bg_ = 20 V, shown in Figure 2(a), the switching current modulates between 60 nA and 10 nA in a periodic fashion, typical of a SQUID interferometer. Since the supercurrent is not zero for any magnetic field, only partial and not complete destructive interference is observed at this back-gate voltage. However, when the back-gate voltage is decreased, as shown in Figure 2(b), the supercurrent modulates completely to zero, and there is full destructive interference. The period of the interference pattern (Δ*B*) is the same for both voltages on the back-gate. The effective area of the SQUID is obtained as *A*_eff_ = Φ_0_/Δ*B*, yielding *A*_eff_ = 26 μm^2^, 1.9 times larger than the geometrical area of the loop, *A*_geo_ = 13.6 μm^2^. This difference can be attributed to the flux-focusing effect of the superconducting strips. Due to the Meissner effect, the strips partially deviate the flux density B⃗ into the loop, effectively increasing the enclosed flux.^31^

**Figure 2:**
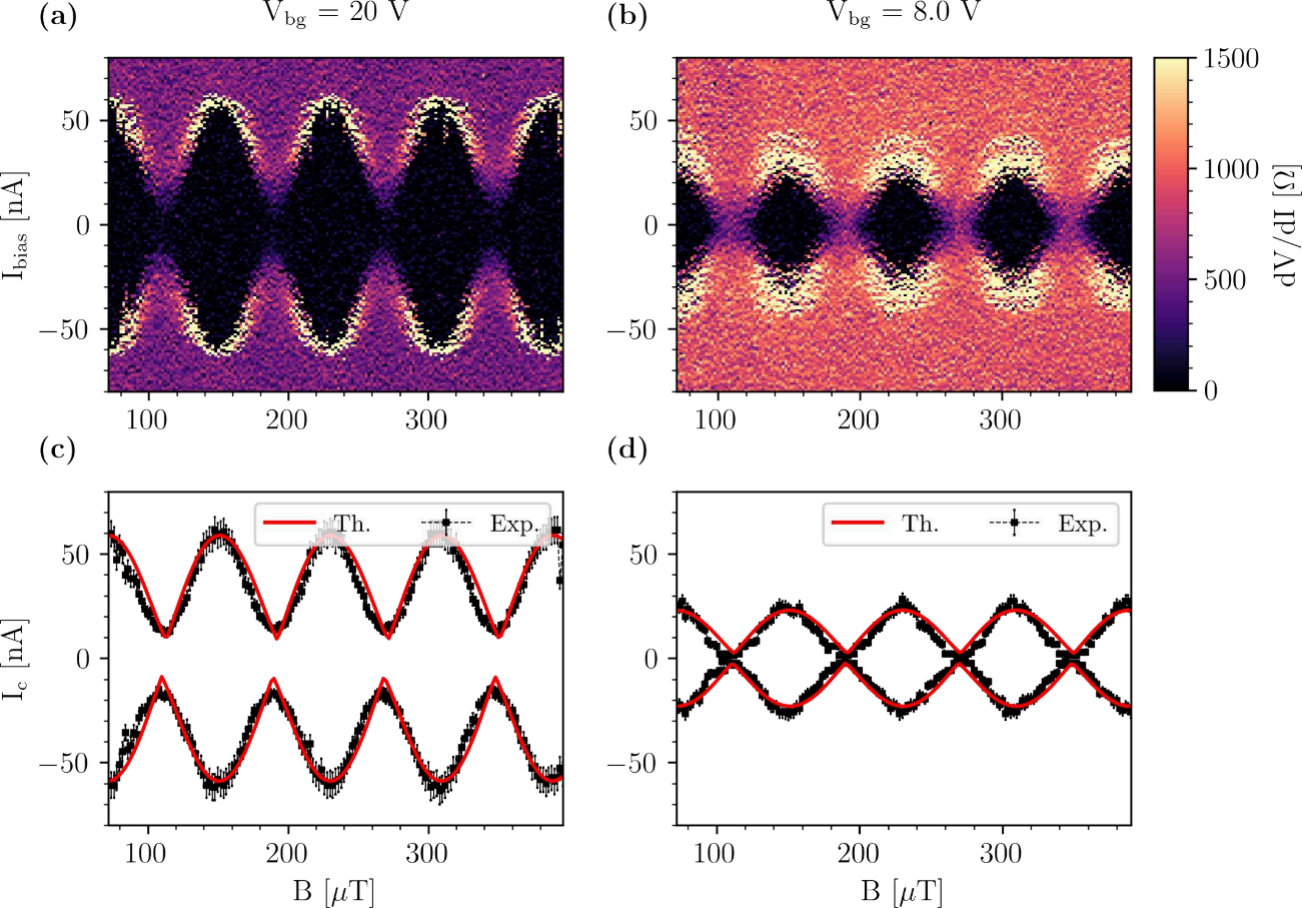
Magnetic field response of the symmetric SQUID. (a), (b) Color maps of the differential resistance *dV*/*dI* as a function of current bias and the applied magnetic field. (a) *V*_bg_ = 20 V, (b) *V*_bg_ = 8 V. The temperature is *T* = 350 mK. (c), (d) Comparison of the critical current between experimental data and theoretical model (see text) for the two back-gate configurations presented in (a) and (b), respectively.

These observations cannot be explained by simply assuming that the two arms of the SQUID support different supercurrents. To understand the observed features, we performed numerical simulations. Details are provided in Section S5 of the Supporting Information. At low energy, the electronic properties of the InSb nanoflags can be described by a two-band model^32^

5 $$H\left(\mathrm{k⃗}\right)=\left(\frac{\hbar^{2}{\mathrm{k⃗}}^{2}}{2m^{*}}-\mu \right)\sigma_{0}-\alpha_{R}k_{y}\sigma_{x}+\alpha_{R}k_{x}\sigma_{y}+E_{B}\sigma_{z}$$

where σ_ν_, ν ∈ {0, *x*, *y*, *z*}, are the Pauli matrices acting on the spin degree of freedom. In the above equation, m* is the effective electron mass in InSb, α_*R*_ the Rashba spin–orbit coupling, μ the chemical potential, and $E_{B}=\frac{1}{2}g\mu_{B}B$ the Zeeman energy. In particular, the adopted material parameters^33^ are m* = 0.014 *m*_*e*_, α_*R*_ = 50 meV nm, and *g* = – 50. The chemical potential is directly proportional to the voltage on the back-gate μ ∝ *V*_bg_ using a simple capacitive model (see Section S6 of the Supporting Information).

The single Josephson junction is described by the Bogoliubov-de Gennes Hamiltonian

6 $$H_{\mathrm{BDG}}\left(x\right)=\left(\begin{array}{ll} H & \Delta \left(x\right)\\ \Delta^{*}\left(x\right) & -THT^{-1} \end{array}\right)$$

where $T=-i\sigma_{y}K$ is the operator implementing time-reversal symmetry and

7 $$\Delta \left(x\right)=\Delta \left[\theta \left(-x\right)+e^{i\phi }\theta \left(x-L\right)\right]$$

with Δ the induced superconducting gap and θ­(*x*) the step function. To compute the Josephson current of the SQUID, we model two JJs in parallel by regularizing eq 6 onto a tight-binding model on a square lattice. Here, we add the orbital effects associated with the magnetic field through the Peierls substitution. Then, we employ the recursive Green’s function formalism^34,35^ to compute the Josephson response (see Section S6 in the Supporting Information).

In Figure 2(c)-(d), we show a comparison of the critical currents resulting from the experimental data extracted from Figure 2(a)-(b), respectively, and the numerical simulations. The simulation parameters can be found in Section S6 of the Supporting Information. The theoretical simulations correctly reproduce the closing of the SQUID pattern (complete destructive interference) at low back-gate voltages. This can be explained as follows: at high back-gate voltages, the CPRs are skewed, resulting in a partially destructive interference; at lower back-gate voltages, on the other hand, the junction transparencies decrease with a correspondingly more sinusoidal behavior of the CPRs. This interpretation is supported by the CPR plots of one of the junctions at zero field reported in Figure 3(a). Here, it can be seen that the CPR at *V*_bg_ = 20 V deviates significantly more from a sinusoidal behavior than at *V*_bg_ = 8 V. To further corroborate this statement, we performed a Fourier analysis of the CPRs as shown in Figure 3(b). Here, the ratio of the spectral weights of the *k*-th harmonics with respect to the fundamental tone is plotted on a logarithmic scale. A significant contribution from higher harmonics is present, especially at high back-gate voltages. This is consistent with a phenomenological relation between the junction transparency and the back-gate voltage derived from the Blonder-Tinkham-Klapwijk (BTK) model,^36^ τ = 1/(1 + *Z* ^2^(*V*_bg_)) (see Sections S5 and S6 of the Supporting Information), whose behavior is reported in the inset to Figure 3(b). We stress that from the above analysis a detailed characterization of the CPR skewness, and its harmonic content, has been achieved.

**Figure 3:**
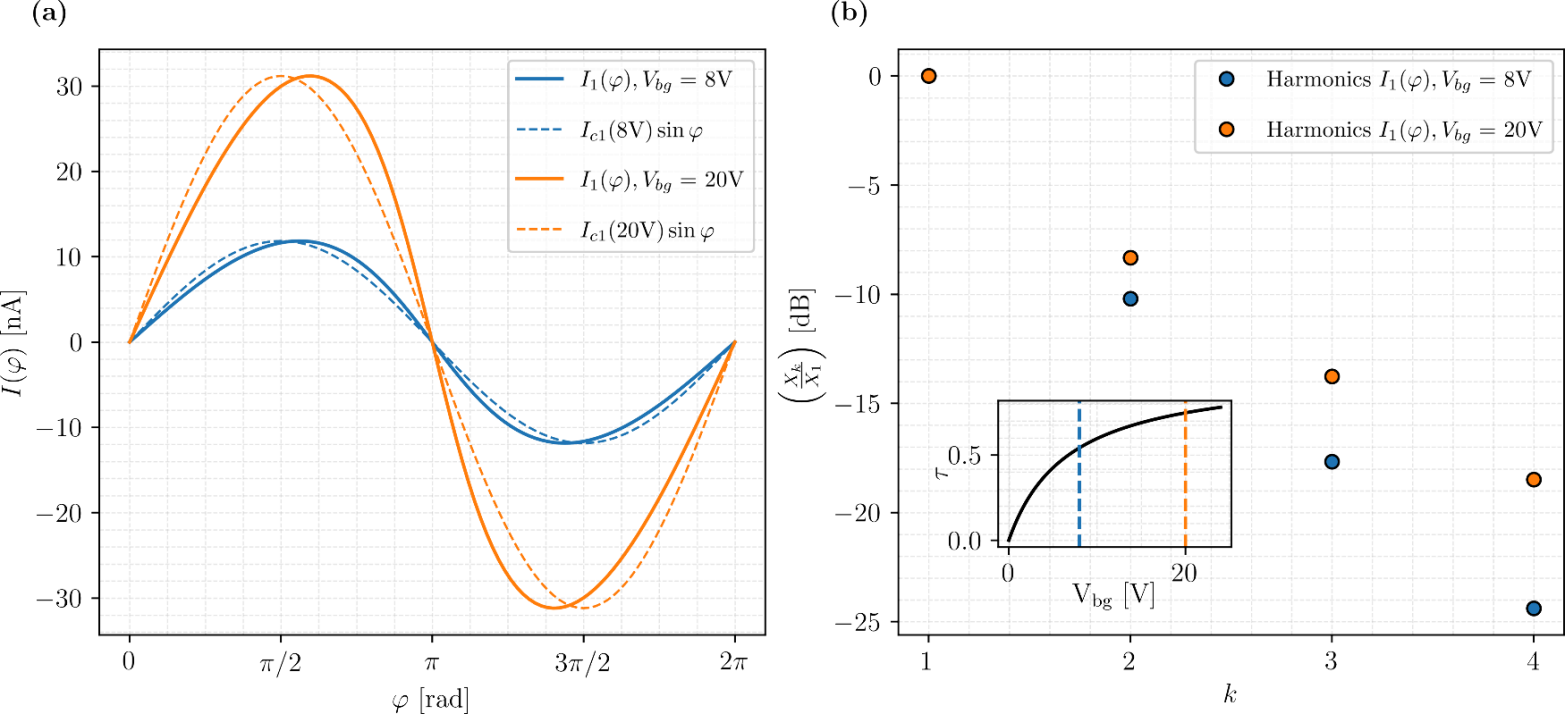
Single junction CPR at zero field. (a) CPR of one of the junctions forming the symmetric SQUID, for *V*_bg_ = 20 V (orange) and *V*_bg_ = 8 V (blue). Dashed lines correspond to fully sinusoidal CPRs with the same critical current amplitudes for comparison. (b) Ratio of the spectral weights *X*_*k*_ with respect to the first harmonic *X*_1_ expressed in dB, for the CPRs in (a). The inset shows a plot of the junction transparency versus back-gate voltage.

A different phenomenology is observed in the asymmetric device of Figure 1(b). Figure 4(a) shows the interference pattern at *V*_bg_ = 12 V, for which characteristic SQUID-type interference is present, and the critical current modulates from *I*_c_ ∼ 100 nA to ∼ 30 nA. The periodicity displayed corresponds to an effective area of *A*_eff_ = 149 μm^2^, 1.26 larger than *A*_geo_ = 118 μm^2^. The flux-focusing factor of this device is slightly lower than that of the symmetric SQUID, which is expected because the asymmetric SQUID has a larger area. Consequently, the ratio of the area of the Nb strips to the loop area is reduced, and as such, the relative amount of magnetic field screened is also diminished.

**Figure 4:**
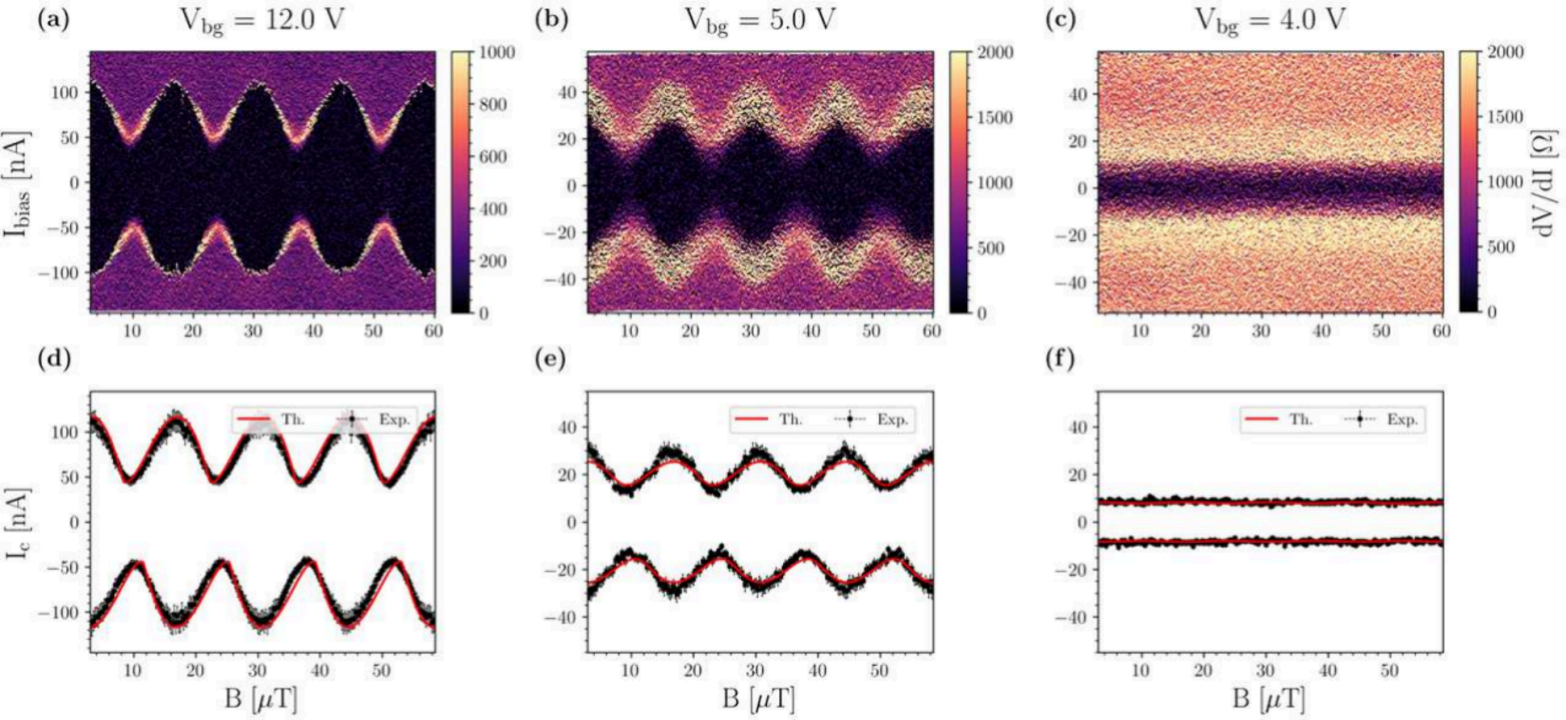
Magnetic field response of the asymmetric SQUID. (a), (b), (c) Color maps of the differential resistance *dV*/*dI* as a function of current bias and the applied magnetic field. (a) *V*_bg_ = 12 V, (b) *V*_bg_ = 5 V, and (c) *V*_bg_ = 4 V. *T* = 350 mK. (d), (e), (f) Comparison of the critical currents between the SQUID experimental data and the theoretical model for the configurations in (a), (b), and (c), respectively.

When *V*_bg_ is reduced, as shown in Figure 4(b) and Figure 4(c), the modulation amplitude of the interference pattern decreases, completely disappearing at *V*_bg_ = 4 V. This last observation indicates that one of the two Josephson junctions is pinched off and below threshold. Thus, the interferometer has only one arm available for transport, and the supercurrent, not enclosing any magnetic flux, does not show a SQUID-like interference. All these features are well captured by the theoretical model described above, adopted for the asymmetric configuration, as shown in panels (d-f) of Figure 4. Again, both junctions forming the SQUID possess skewed CPRs at high back-gate voltages. It is worth highlighting that the SQUID patterns shown in Figure 4(a-b) do not show complete destructive interference due to the asymmetry in the geometry of this SQUID device. Section S7 of the Supporting Information shows more interference patterns for other back-gate voltages.

Upon closer inspection of Figure 4(a), it can be seen that there is a difference in critical current when sweeping the current bias up (*I*_c+_) or down (|*I*_c‑_|). The magnetic fields at which *I*_c+_ and |*I*_c‑_| have minima are not the same. This behavior is characteristic of nonreciprocal transport, showing the so-called Josephson diode effect.^28,37^ Consequently, at a fixed magnetic field there is a range of current bias values where the SQUID is superconducting in one current bias direction and dissipative in the other. This feature is related to the asymmetric configuration, and can be quantified by the rectification coefficient η = (*I*_c+_ – |*I*_c‑_|)/(*I*_c+_ + |*I*_c‑_|) which reaches 6% here. The rectification coefficient is also found to be gate-tunable and goes to zero for *V*_bg_ = 4 V. This feature connects the Josephson diode effect to the semiconducting nanoflags and excludes other sources of superconducting diode, such as vortices or superconductor screening currents.^38–40^ We note that consistent results were recently found in InSb nanosheet interferometers.^37^ Additional details on the Josephson diode effect for this SQUID device can be found in Section S8 of the Supporting Information, while Section S9 shows Fraunhofer-like interference patterns in a wider range of magnetic field values.

SQUIDs in the nonhysteretic regime are commonly employed as flux-to-voltage transducers to measure small variations in the magnetic field. To test the performance of these nanoSQUIDs based on InSb nanoflags as a magnetometer, they are current biased in the dissipative state, at a working point where the *V* – *B* response is optimal. We have obtained very similar results for the two geometries. Here we discuss only the asymmetric SQUID. Details on symmetric SQUIDs can be found in Section S10 of the Supporting Information. In Figure 5(a) we report the *V* – *B* curves of the asymmetric SQUID for different values of current bias at *V*_bg_ = 18 V. For a current bias of 100 nA, a modulation amplitude of about 20 μV is obtained. We use the voltage responsivity to characterize the voltage response of the device. This quantity, which is a standard figure of merit of a magnetometer, is defined as

8 $$V_{\Phi }={\frac{\partial V}{\partial \Phi }|}_{I_{\mathrm{bias}}}$$

and can be used to relate the voltage noise amplitude $S_{V}^{1/2}$ to the magnetic flux noise amplitude $S_{\Phi }^{1/2}$ , using $S_{V}^{1/2}=V_{\Phi }\cdot S_{\Phi }^{1/2}$ .^2^ These quantities are known to be a function of frequency. In particular, the voltage noise amplitude tends to increase as the frequency is decreased, and the 1/*f* noise contribution dominates the white noise spectrum typical of thermal noise.^3,41^ DC transport measurements reported here can provide an estimate of the magnetic flux noise at low frequency, *f* < 10 Hz, limited by the multimeter integration time.

**Figure 5:**
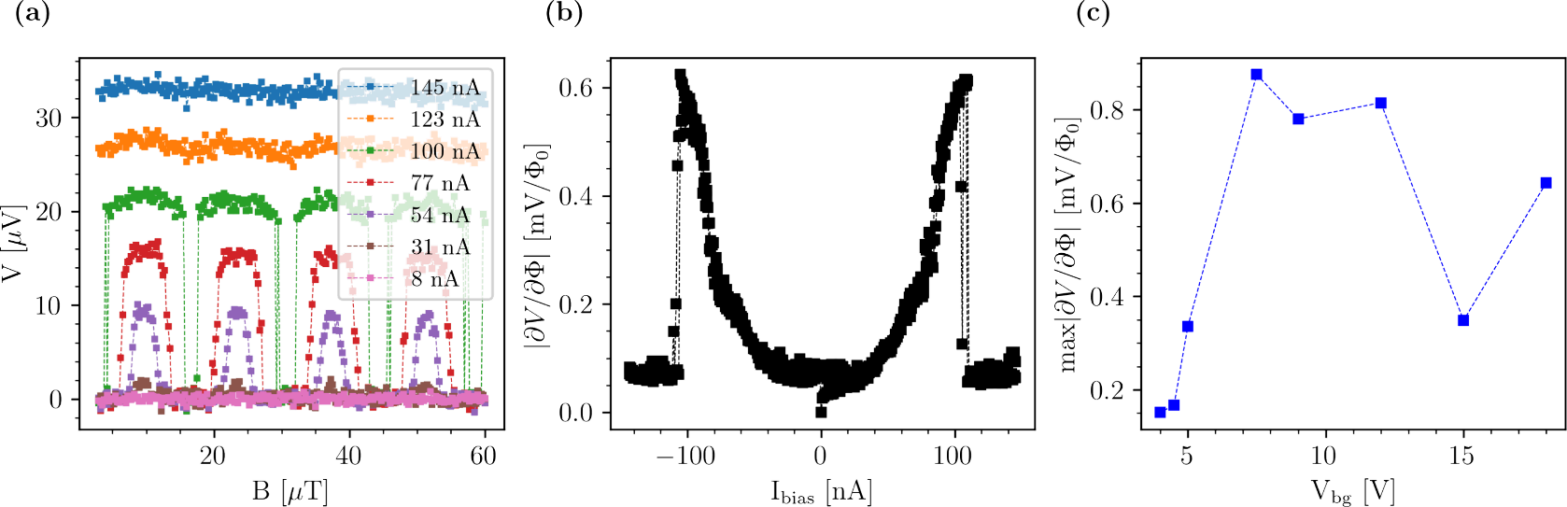
Transfer function characteristics of the asymmetric SQUID. *T* = 350 mK, *V*_*bg*_ = 18 V. (a) *V*–*B* characteristics for different values of current bias. *V*_*bg*_ = 18 V. (b) Voltage responsivity as a function of current bias. (c) Maximum responsivity as a function of back-gate voltage.

In Figure 5(b) we show the measured voltage responsivity as a function of the current bias for *V*_bg_ = 18 V. A maximum of *V*_Φ_ = 0.6 mV /Φ_0_ is obtained. This value can be further improved by tuning the back-gate, as shown in Figure 5(c). A maximum of 0.9 mV /Φ_0_ is found for *V*_bg_ = 7.5 V. To convert this quantity to magnetic flux noise, an evaluation of the voltage noise of the experimental setup is needed. In our setup at *T* = 350 mK, the main source of noise is the input noise of the room-temperature preamplifier, $S_{V,\mathrm{preamp}}^{1/2}=$ 4nV $/\sqrt{\mathrm{Hz}}$ , which finally gives $S_{\Phi }^{1/2}=4.4\times 10^{-6}{\;\Phi }_{0}$ $/\sqrt{\mathrm{Hz}}$ . This value is consistent with the performance of commercially available superconducting magnetometers^42^ and is comparable to those documented in the literature, encompassing both all-metallic configurations^6,43–47^ and InAs-based interferometers.^48,49^ This comparison underscores the superior quality of these devices, especially considering the ultracompact dimensions ( $∼10\mu m^{2}$ ) of these nanoSQUIDs. With further optimization, the sensitivity of SQUIDs fabricated from InSb nanoflag Josephson junctions has the potential to be enhanced for application in magnetometry.

In this study, superconducting quantum interference devices realized using InSb nanoflag Josephson junctions have been thoroughly investigated. Two distinct geometries were characterized, in which the dual junctions forming the SQUID are arranged in either symmetric or asymmetric configurations. An appropriate theoretical framework adequately accounts for all observed phenomena. Specifically, important information on the CPR of InSb nanoflag based JJ has been obtained. Interestingly, the higher harmonic content of these junctions, which yields skewed current-phase relations (CPRs), has been quantified. It was demonstrated that the transparency of the junction can be modulated by a back-gate voltage, leading to partial destructive interference in the symmetric configuration at elevated voltages and complete destructive interference at reduced voltages. We note that the asymmetric configuration exhibits nonreciprocal supercurrent transport with a diode efficiency of ∼6% , which is consistent with a recent complementary study.^37^ This observation further corroborates the higher harmonic content and superior quality of these junctions. Additionally, the SQUID performance has been evaluated, particularly their low-frequency flux-to-voltage response, highlighting their potential applicability as novel nanoscale magnetometers.

## Supplementary Material



## Notes

Fabrication methods, measurement methods, determination of the threshold voltage, inductance estimation, modeling of a single Josephson junction, simulation of the SQUID setup, additional data, Josephson diode effect in the asymmetric SQUID, Fraunhofer-like interference, and magnetometer response for the symmetric SQUID (PDF)
