# Supplementary material for: Unveiling the Current-Phase Relationship of InSb Nanoflag Josephson Junctions Using a NanoSQUID Magnetometer
Source: Nano Lett. 2025 Sep 10;25(39):14412–9. doi: 10.1021/acs.nanolett.5c03765 (PMC12492403; doi:10.1021/acs.nanolett.5c03765)
Supplement: Supplementary file 1 [file nl5c03765_si_001.pdf]

Supporting Information for "Unveiling the current-phase-relationship of InSb nanoflag Josephson junctions using a nanoSQUID magnetometer"

Andrea Chieppa,[1] Gaurav Shukla,[1] Simone Traverso,[2,3] Giada Bucci,[1] Valentina Zannier,[1] Samuele Fracassi,[2,3] Niccolo Traverso Ziani,[2,3] Maura Sassetti,[2,3] Matteo Carrega,[3,*] Fabio Beltram,[1] Francesco Giazotto,[1] Lucia Sorba,[1] and Stefan Heun[1,†]

[1]*NEST, Istituto Nanoscienze-CNR and Scuola Normale Superiore, Piazza San Silvestro 12, 56127 Pisa, Italy*

[2]*Dipartimento di Fisica, Università di Genova, Via Dodecaneso 33, 16146 Genova, Italy*

[3]*CNR-SPIN, Via Dodecaneso 33, 16146 Genova, Italy*

(Dated: September 4, 2025)

Abstract

This file contains additional information on the fabrication methods, supporting experimental data, and details on the theoretical simulations.

S1. FABRICATION METHODS

The fabrication process of the SQUIDs begins with the as-grown sample of InSb nanoflags, which are attached to the Indium Phosphide stems [1]. The initial step involves transferring the free-standing structures onto a highly conductive p-type Si(100) substrate, serving as a global back-gate. A 285 nm thick SiO_2 layer covers the Si substrate, acting as a dielectric.

A standard electron beam lithography (EBL) technique is used for fabricating the Nb contacts. First, 270 nm of AR 679.04 resist is spin-coated at 4000 rpm for 1 minute, followed by baking the sample at 170 °C for 90 s. EBL is performed at 20 kV accelerating voltage of electrons, 10 µm aperture, $\approx$ 33 pA current, and 290 µC cm^{-2} dose using a 200 $\times$ 200 µm^2 write field. After EBL, the pattern is developed in AR 600-56 for 1 minute, followed by rinsing in IPA for 30 seconds prior to drying with N_2 flux. The developed pattern is then exposed to O_2 plasma (15 W for 75 seconds) for descum to remove any residual resist in the pattern.

Prior to sputtering Niobium on the EBL-patterned sample, to achieve ohmic contacts [2], the exposed area of the InSb nanoflags is passivated by immersing the sample in $(NH_4)_2S_x$ (290mM$(NH_4)_2S$ and 330mM S in deionized water) at 45 °C for 60 seconds, followed by cleaning in deionized water for 30 seconds prior to drying with N_2 flux.

Sputtering of 180 nm of Niobium is performed at 150 W for 240 seconds (at a rate of 7.5 Å/s) at a base pressure of 8×10^{-8} mbar and a working pressure of 5×10^{-3} mbar in the presence of Ar. The sputtered sample is then kept in acetone overnight for the lift-off process, followed by cleaning in IPA for 30 seconds.

After completing the fabrication process, the sample chip is glued to a dual-in-line chip carrier using highly conductive silver paste, which enables the operation of the back-gate. The individual SQUIDs are then connected to the chip carrier by Aluminum wire bonding.

* matteo.carrega@spin.cnr.it

† stefan.heun@nano.cnr.it

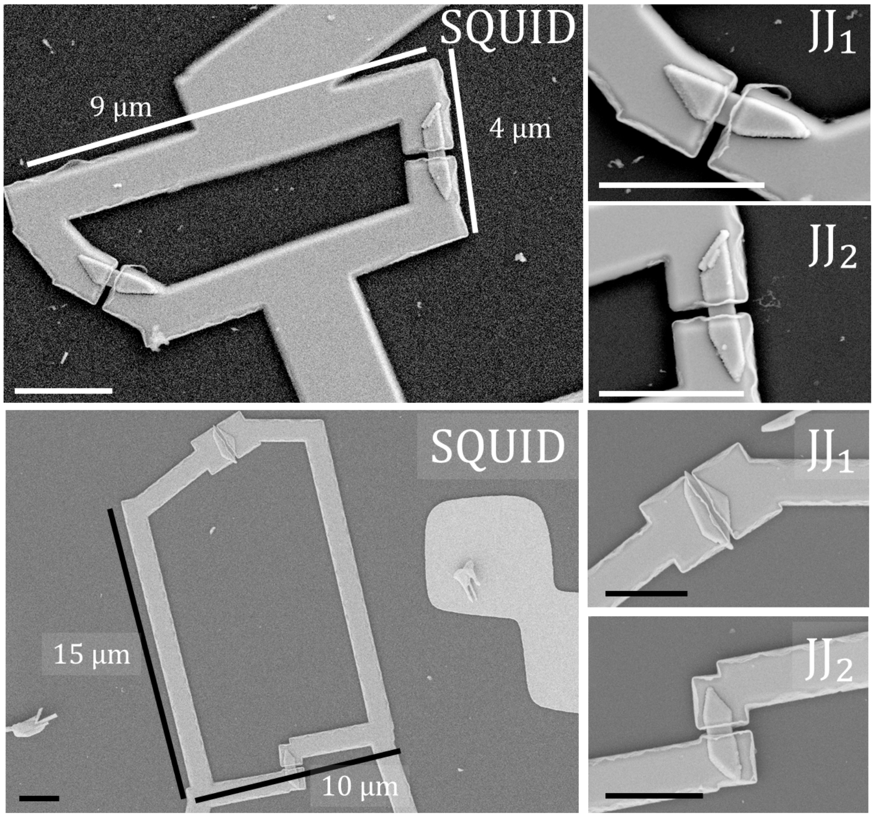

FIG. S1. SQUID in (top) symmetric and (bottom) asymmetric geometry. A zoom on both Josephson junctions is displayed in the right panels. Scale-bars in the lower left corner of each panel indicate 2 µm.

The two geometries introduced in the main text are displayed in the scanning electron micrographs in Fig. S1. The geometrical parameters are given in Table S1.

	L1	L2	W1	W2	A_{JJ1}	A_{JJ2}	A_{loop}
Symmetric SQUID	200	200	380	380	0.11	0.11	13.6
Asymmetric SQUID	180	190	1700	530	0.44	0.14	118

TABLE S1. Geometrical parameters of the devices presented in this work. Subscript i refers to the Josephson junction i, shown in Fig. S1 (a) and (b). Lengths in nm, areas in µm^2. For the area of the junctions, A_{JJi}, twice the London penetration depth of Niobium has been included ($\lambda_{\mathrm{L}} = 43\,\mathrm{nm}$ [3]).

S2. MEASUREMENT METHODS

Magneto-transport measurements have been performed in an Ice-Oxford dry cryostat, with a continuous-mode base temperature of $T = 350\,\mathrm{mK}$. The conductance measurements shown in Fig. 1(c) and (d) of the main text are performed at $T = 2\,\mathrm{K}$ with an AC current bias setup, using two SR-830 Lock-In Amplifiers. The back-gate and the solenoid superconducting magnet are powered with two Keithley model 2400B Source Measure Unit. The voltage-current characteristics were acquired in current bias feeding a current with a Yokogawa GS200 generator and employing two Stanford Research preamplifiers, model SR560 and SR570, measuring the amplified signals with two Agilent 34401A multimeters.

S3. DETERMINATION OF THE THRESHOLD VOLTAGE

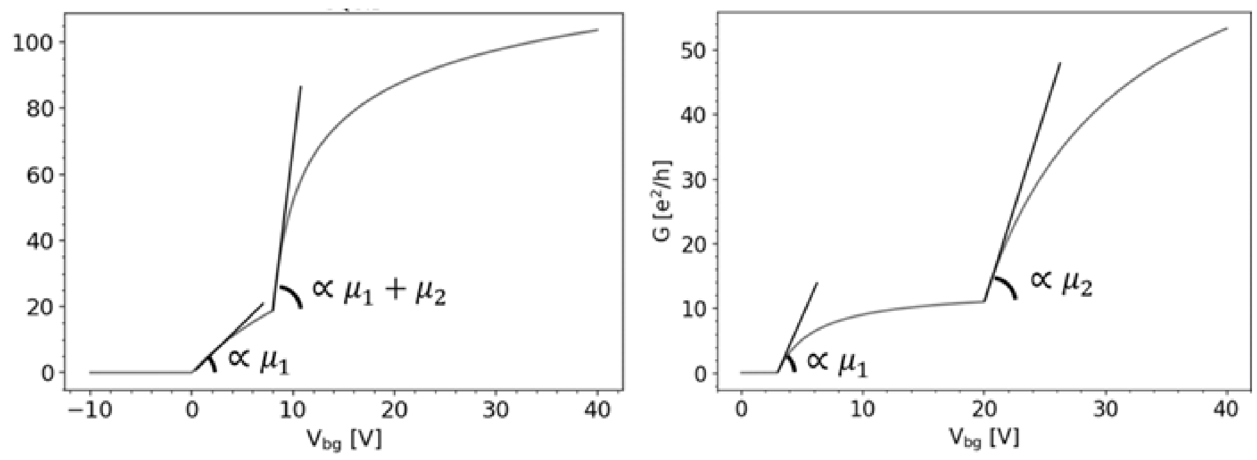

FIG. S2. Model of the conductance of a SQUID. G vs. V_{bg} curves obtained using the model of the conductance of a SQUID given in Eq. (1) of the main text. Left: The parameters used are $V_{th,1} = 0$ V, $V_{th,2} = 8$ V, $\mu_1 = 4000$ cm^2(Vs)$^{-1}$, $\mu_2 = 10000$ cm^2(Vs)$^{-1}$, $R_{c1} = R_{c2} = 200$ Ω. For a small difference in the voltage thresholds, the conductance at the upper threshold has a slope that is proportional to the sum of the effective mobilities. Right: The parameters used are $V_{th,1} = 3$ V, $V_{th,2} = 20$ V, $\mu_1 = 5000$ cm^2(Vs)$^{-1}$, $\mu_2 = 2000$ cm^2(Vs)$^{-1}$, $R_{c1} = 1000$ Ω, $R_{c2} = 200$ Ω. For very different voltage thresholds, the slope of the conductance at the upper threshold is proportional to the effective mobility of the corresponding junction.

In a generic SQUID setup, when a semiconducting channel opens due to an increase of the back-gate voltage beyond a certain threshold voltage, this is represented by a positive contribution to the slope of the G vs. V_{bg} curve at that threshold. This behavior is illustrated for two representative SQUIDs in Fig. S2. Consequently, variations in the slope of the G vs. V_{bg} curve serve as indicators for identifying the threshold voltage V_{th} associated with each junction comprising the SQUID.

Here, to quantitatively identify the threshold voltages $V_{th,1}$ and $V_{th,2}$, a least-squares fitting method has been chosen. We have opted for this method since it allows for a more unbiased determination of the threshold voltages. We utilize the *curve_fit* routine from the Python-based SciPy package. The model, described in Eq. (1) of the main text, uses the electrical parallel of two Josephson junctions, each defined by the following conductance

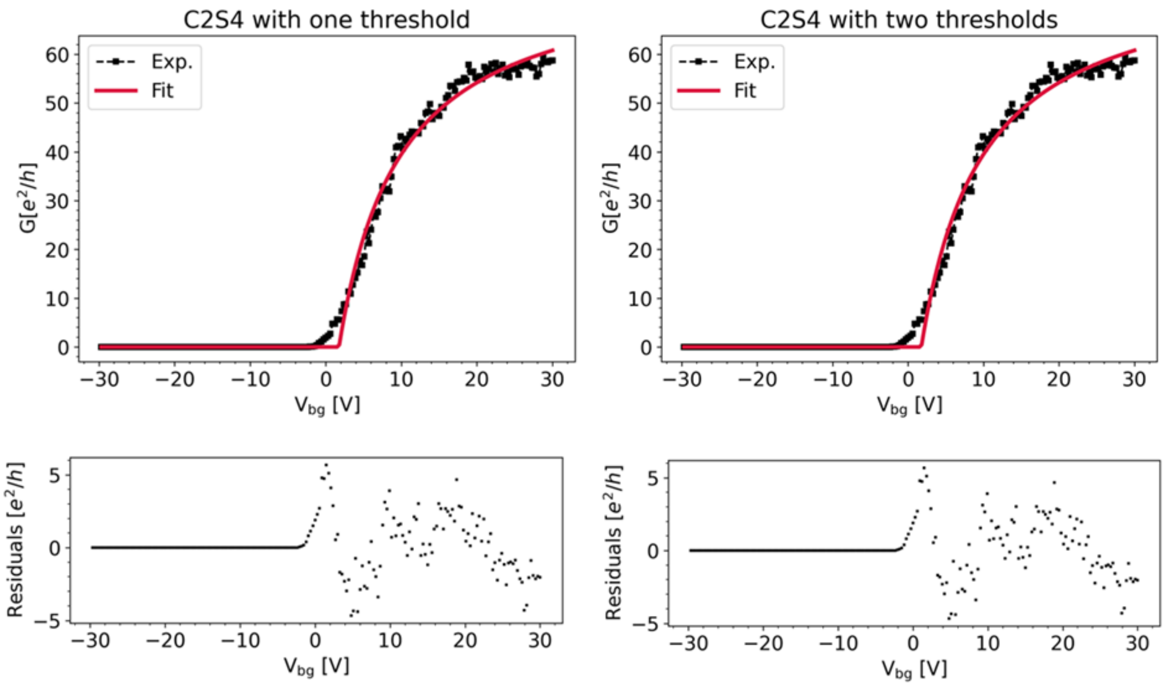

FIG. S3. Top row: Experimental conductance (black squares) and fit (red solid line). Bottom row: residuals, defined as experimental data minus model calculated for the optimal parameters. Left: Optimal parameters found: $V_{th} = 1.7 \pm 0.1$ V, $\mu = 8586 \pm 280$ cm^2(Vs)$^{-1}$, $R_c = 330 \pm 5$ Ω. Right: $V_{th,1} = 1.7$ V, $V_{th,2} = 1.8$ V, $\mu_1 = 8561$ cm^2(Vs)$^{-1}$, $\mu_2 = 8609$ cm^2(Vs)$^{-1}$, $R_c = 330$ Ω.

$G_{JJ,i}$:

$$\frac{1}{G_{JJ,i}} = \left(\frac{C_{ox}W}{L}\mu\left(V_{bg} - V_{th}\right)\right)^{-1} + 2R_c, \tag{1}$$

where $C_{ox} = \epsilon/t$ is the capacitance per unit area, W is the channel width (that we assume to be equal to the nanoflag width), L is the channel length (that we assume to be equal to the distance between the electrodes), and μ is an effective mobility near the channel opening threshold V_{th}. A contribution of the contact resistance R_c of the two InSb/Nb interfaces is also included as a resistance in series with the junction.

We first fitted the symmetric curve with a model where each JJ is independent (5 parameters: $V_{th,1}$, $V_{th,2}$, μ_1, μ_2, R_c). As shown in Fig. S3, the best fit results in $V_{th,1} \sim V_{th,2}$ and $\mu_1 \sim \mu_2$. In other words, the numerical problem is ill-conditioned because the parameters are not independent. Repeating the procedure with the constraint that $V_{th,1} = V_{th,2}$ and $\mu_1 = \mu_2$ (reducing the number of parameters to three) yields the same quantitative results, highlighting that we cannot distinguish between the two junctions. This is in stark contrast

to the data shown for the asymmetric SQUID, where two thresholds are visible.

Finally, our analysis is consistent with the fact that for the symmetric SQUID, we observe a fully developed SQUID pattern for all back gate voltages, as long as a supercurrent is observable.

Regarding the small difference between the threshold voltages observed in Fig. 1 and Fig. 4 of the main text, we first note that the data in Fig. 1 were measured at 2 K, whereas the data in Fig. 4 were measured at 350 mK. Besides, we attribute the shift to hysteresis in the measurement involving the back gate. This is a common problem encountered when using thermally grown silicon dioxide that presents charge traps. We minimize its influence by a strict gating protocol (we always arrive from higher gate voltages to a desired set point), but still, once a specific voltage is applied to the global back gate, the effective potential applied to the semiconducting nanoflag slowly drifts with time and relaxes to an asymptotic value (time scales ~ 0.5 h). This is a small but well-understood effect. Here, it is noticeable because the data points for the back gate sweep were measured immediately after a specific back gate voltage had been set, whereas for the SQUID maps, the back gate was set some time before the map measurement began.

S4. INDUCTANCE ESTIMATION

The magnetic flux enclosed in the SQUID loop is the sum of an external applied flux and an induced flux. The latter is due to the self-inductance L, which is the sum of the geometrical contribution L_{geo} and the kinetic contribution L_{kin}, i.e., $L = L_{geo} + L_{kin}$. When the SQUID supports supercurrent transport, a finite value of L shifts the working point in the magnetic flux by LI_{circ}, where I_{circ} is the supercurrent circulating in the loop. To understand the relevance of this effect, the inductance parameter $\beta_L = 2\pi\frac{LI_c}{\Phi_0}$ is a useful quantity. Usually, when β_L ranges from 10^{-1} to 1, the interpretation of an interference pattern must explicitly take into account a finite L, and precise calculations of it are needed. This often goes through numerical simulations and finite element methods. However, a prior estimate of L is needed to understand if it produces any significant effect.

This is done calculating the geometrical contribution, L_{geo}, under the assumption of a rectangular loop (sides l, w) with a circular cross section (radius r_w) [4]:

$$L_{geo} = \frac{\mu_0}{\pi}\left[-(l-r_w)\sinh^{-1}\frac{l-r_w}{w-r_w} - (w-r_w)\sinh^{-1}\frac{w-r_w}{l-r_w} + (l-r_w)\sinh^{-1}\frac{l-r_w}{r_w} + \right.$$
$$+ (w-r_w)\sinh^{-1}\frac{w-r_w}{r_w} + r_w\sinh^{-1}\frac{r_w}{w-r_w} + r_w\sinh^{-1}\frac{r_w}{l-r_w} + 2\sqrt{(l-r_w)^2+(w-r_w)^2}$$
$$\left. -2\sqrt{(w-r_w)^2+(r_w)^2} - 2\sqrt{(l-r_w)^2+(r_w)^2} - 2r_w\ln\left(1+\sqrt{2}+2\sqrt{2r_w}\right)\right] \quad (2)$$

For instance, using $l = 9\,\mu\mathrm{m}$, $w = 4\,\mu\mathrm{m}$, and $r_w = 0.36\,\mu\mathrm{m}$ for the symmetric SQUID leads to $L_{geo} = 9.7\,\mathrm{pH}$.

The kinetic contribution L_{kin} arises due to the kinetic energy of Cooper pairs, and for a superconducting wire of length l, width w, and thickness t, can be estimated with the following formula [5, 6]:

$$L_{kin} = \frac{\mu_0\lambda_L^2 l}{wt} \quad (3)$$

In practical situations, this contribution is relevant only when the superconducting strips have thickness comparable to or smaller than the London penetration depth λ_L. In our case, the strips have thickness $t = 150\,\mathrm{nm}$, larger than $\lambda_\mathrm{L} = 43\,\mathrm{nm}$ [3], and an explicit calculation for the symmetric SQUID gives $L_{kin} = 0.3\,\mathrm{pH}$.

For this device, a total inductance of $10\,\mathrm{pH}$ is found, which together with the upper limit on $I_c \sim 100\,\mathrm{nA}$ ultimately leads to a β_L of the order of 10^{-3}. The same procedure for the asymmetric device gives also a β_L of the order of 10^{-3}. Hence, we conclude that corrections

to the magnetic flux owing to the self-inductances of the loops can be considered negligible.

S5. MODELING OF A SINGLE JOSEPHSON JUNCTION

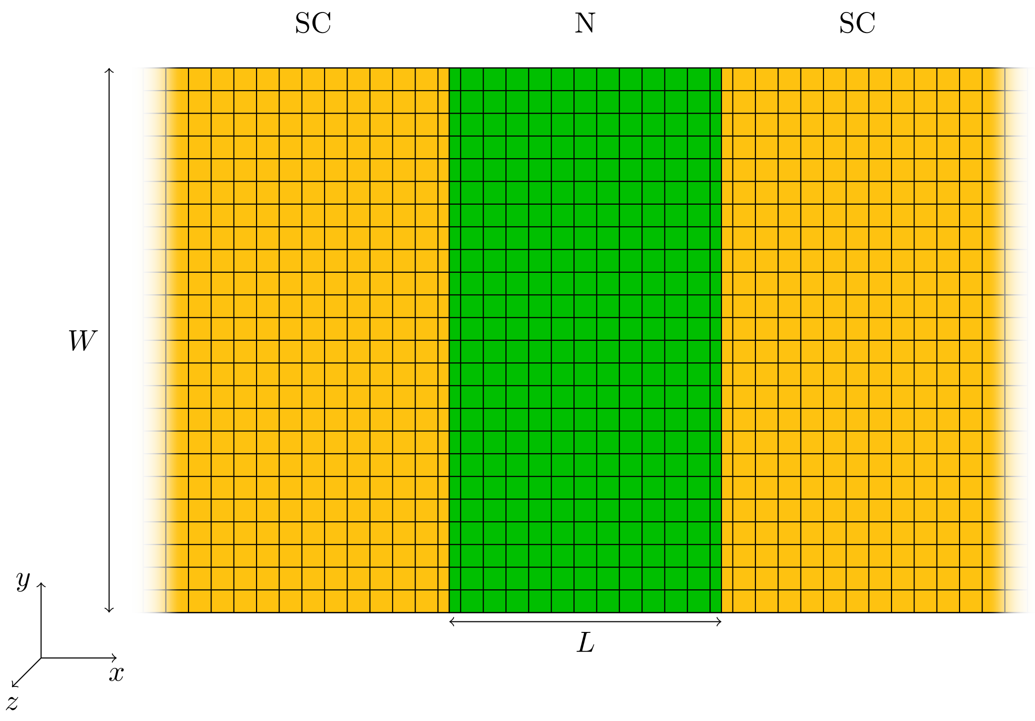

FIG. S4. Scheme of a planar Josephson junction, overlaid the discretized square lattice and located in the $x-y$ plane. The normal region is colored in green, while the superconducting leads are in yellow.

We consider a planar Josephson junction in the $x-y$ plane and extending along the x-direction, with the left and right leads obtained by proximitizing the InSb nanoflag with a conventional s-wave superconductor. We denote by L the junction length, and by W its width. Furthermore, we denote by (x_0, y_0) the coordinates of the site at the bottom left corner of the scattering region.

First, to obtain a tight-binding Hamiltonian for the InSb nanoflag in the normal region, we regularize the continuum Hamiltonian of the main text [Eq. (5)] on a square lattice with lattice constant a. The resulting $\vec{k}$-space Bloch Hamiltonian is given by

$$\mathcal{H}(\vec{k}) = \{(4t-\mu) - 2t[\cos(k_x)+\cos(k_y)]\}\sigma_0 - 2\mathcal{E}_R\sin(k_y)\sigma_x + 2\mathcal{E}_R\sin(k_x)\sigma_y + \mathcal{E}_B\sigma_z, \quad (4)$$

where σ_0 is the 2×2 identity matrix, and $\sigma_i,\ i=x,y,z$ are the Pauli matrices acting on the spin degree of freedom. Here the Bloch momentum is adimensional, $t = \frac{\hbar^2}{2m^* a^2}$ parametrizes the first neighbor hopping, and m^* is the electron effective mass. Moreover, $\mathcal{E}_B$ and $\mathcal{E}_R$ are

Parameter	symbol	value
effective mass	m^*	$0.014\ m_E = 1.27 \cdot 10^{-32}$ kg
lattice discretization	a	10nm
gyromagnetic factor	g	-50
Rashba coupling	α_R	50 meV nm

TABLE S2. Numerical values of the parameters used in the Hamiltonian for the simulation of InSb nanoflags.

energy scales associated to the magnetic field and to the Rashba spin-orbit coupling. They are defined as

$$\mathcal{E}_B = \frac{1}{2} g \mu_B B, \tag{5}$$

$$\mathcal{E}_R = \frac{\alpha_R}{2a}. \tag{6}$$

with g the gyromagnetic factor, μ_B the Bohr magneton, and α_R the Rashba coupling. The parameter values used in the simulation are listed in Table S2.

We now write down the real space tight-binding Hamiltonian for the junction. Starting from the Fourier transform of Eq. (4), we add magnetic field related orbital effects and random disorder to the normal region, s-wave superconducting pairing to the proximitized leads, and potential barriers at the interfaces. We denote by $\tilde{L} = L/a$ and $\tilde{W} = W/a$ the number of sites along the horizontal and vertical dimension of the scattering region, with $a = 10$ nm the discretization lattice parameter used in the simulations. We associate the lattice indices $(\ell, j) = (0, 0)$ to the bottom-left site at coordinate (x_0, y_0). Then, the real space Hamiltonian for the junction can be written as $H = H_0 + H_{\mathrm{SC}}$, where

$$H_0 = \sum_{\ell,j} \Psi^\dagger_{\ell,j} H_{\mathrm{o.s.}}(\ell, j) \Psi_{\ell,j} + (\Psi^\dagger_{\ell,j+1} V_y \Psi_{\ell,j} + \mathrm{h.c.}) + (\Psi^\dagger_{\ell+1,j} V_x(\ell, j) \Psi_{\ell,j} + \mathrm{h.c.}), \tag{7}$$

$$H_{\mathrm{SC}} = \Delta_\ell c^\dagger_{\uparrow\ell,j} c^\dagger_{\downarrow\ell,j} + \mathrm{h.c.}, \tag{8}$$

with the spinor $\Psi^T_{\ell,j} = (c_{\uparrow\ell,j}, c_{\downarrow\ell,j})$ collecting the operators $c_{\uparrow\ell,j}$ and $c_{\downarrow\ell,j}$, which in turn destroy an electron with spin up and spin down, respectively, at the site indexed by (ℓ, j).

Let us break down the various terms appearing in the Hamiltonian, starting from H_{SC}.

This accounts for the induced s-wave superconductivity in the leads, and Δ_ℓ is given by

$$\Delta_\ell = \begin{cases} \Delta & \ell < 0 \\ 0 & 0 \leq \ell < \tilde{L} \\ \Delta e^{i\phi} & \ell > \tilde{L} \end{cases}, \tag{9}$$

with Δ the induced gap amplitude and ϕ the superconducting phase difference between the two leads.

Regarding H_0, instead, the matrices $H_{\text{o.s.}}$, V_x, and V_y have the explicit expressions

$$H_{\text{o.s.}}(\ell, j) = (4t - \mu + \omega_{\ell,j} + \mathcal{U}_\ell)\sigma_0 + \frac{1}{2} g\mu_B B_\ell \sigma_z, \tag{10}$$

$$V_x(\ell, j) = [-t\sigma_0 + i\mathcal{E}_R\sigma_y] e^{-i\frac{e}{\hbar}B_\ell(ja - y_0)a}, \tag{11}$$

$$V_y = -t\sigma_0 - i\mathcal{E}_R\sigma_x. \tag{12}$$

The magnetic field spatial dependence is given by

$$B_\ell = \begin{cases} 0 & \ell < 0 \\ B & 0 \leq \ell < \tilde{L} \\ 0 & \ell > \tilde{L} \end{cases}, \tag{13}$$

that is, the magnetic field is assumed to be uniform in the normal region and zero in the leads. The orbital effects are included via the Peierls phase in V_x, which results from the choice of the Landau gauge $\vec{A} = (-By, 0, 0)$ for the vector potential.

The on-site energies $\omega_{\ell,j}$ are added to account for possible substrate-induced disorder in the normal region ($\omega_{\ell,j} = 0$ for $\ell < 0$ or $\ell \geq \tilde{L}$), and are randomly extracted from the interval $[-V_{\text{dis}}/2, V_{\text{dis}}/2]$ ($V_{\text{dis}} > 0$) with an uniform distribution.

Finally, the term $\mathcal{U}_\ell = \mathcal{U}(\delta_{0,\ell} + \delta_{\tilde{L}-1,\ell})$ models two potential barriers, placed on the first and last column of sites of the normal region, respectively. The barriers are added to tune the transparency at the NS interface, which according to the Blonder-Tinkham-Klapwijk (BTK) model [7] is defined as

$$\tau = \frac{1}{1 + Z^2}, \tag{14}$$

with $Z = m^*\mathcal{U}a/(\hbar^2 k_F)$ and k_F the Fermi momentum. If we assume a parabolic dispersion of the bands (which is indeed the case at low energy) and $\mu = \hbar^2 k_F^2/(2m^*)$, then we have $Z = \sqrt{\frac{m^*}{2\mu\hbar^2}}\mathcal{U}a$.

Given the tight-binding Hamiltonian of both the leads and the scattering region in Eq. (7), the equilibrium Josephson supercurrent is computed through the recursive Green's function approach [8, 9]. Concerning the surface Green's functions of the semi-infinite uncoupled leads, these are computed via the infinite recursive Green's function method [10].

S6. SIMULATION OF THE SQUID SETUP

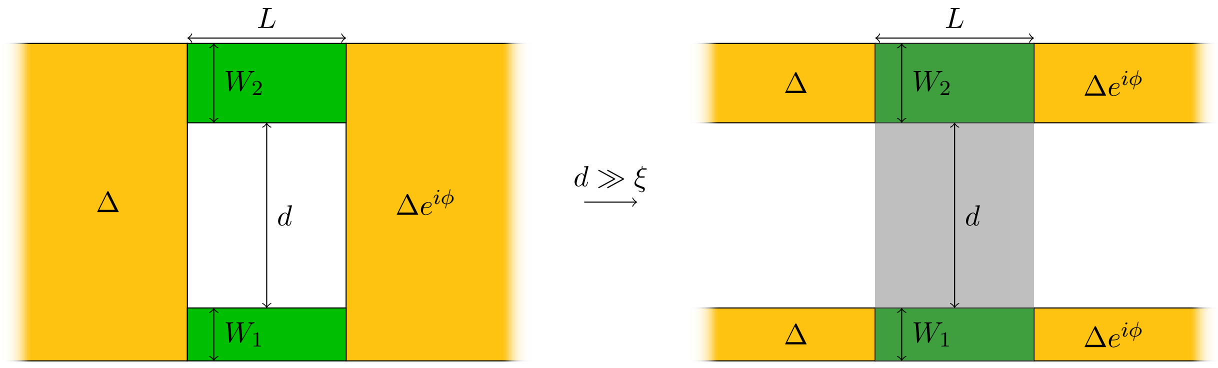

FIG. S5. Scheme of the SQUID setup considered for the simulations. The SQUID geometry on the left, with the superconducting leads covering the left and right ends of the two nanoflags, can be simulated as two independent junctions whenever the separation between the flags is much larger than the superconducting coherence length. This effective setup is shown on the right. The transparent gray overlay covering the normal regions of the flags and the region between them indicates the region where the magnetic field is present.

We model the SQUID setup as shown in Fig. S5. Here, two junctions of width W_1 and W_2 are parallel to each other and have the same length L. We denote the separation between the two junctions by d. Although the superconducting loop geometry is markedly different with respect to that of the actual experimental devices, this is not an issue. Indeed, the response of a SQUID should not change due to deformations of the superconducting loop, as long as the magnetic flux piercing through it is kept constant.

Furthermore, to numerically compute the supercurrent in the SQUID configuration we resort to the following approximation: since the two junctions forming the SQUID are separated by a distance d much larger than the coherence length of the superconductor ($d \gg \xi$), they can be regarded as effectively decoupled (see the right part of Fig. S5). Thus, we actually compute the supercurrent of two independent JJs having same length L, widths W_1 and W_2, and, most importantly, presenting the same superconducting order parameter on the left (Δ) and right ($\Delta e^{i\phi}$) sides, respectively. Crucially, the spatial separation between the junctions (and consequently the field flux in the loop area) is fully accounted for by the y-dependence in the Peierls substitution, correctly yielding the expected interference pattern. In fact, if we fix $y = 0$ at the bottom of the lower junction, then the bottom of the upper one

Parameter	Symmetric SQUID	Asymmetric SQUID
L	200nm	180nm
W_1	380nm	530nm
W_2	380nm	1700nm
Δ	280μeV	300μeV
$\mathcal{U}_1$	55meV	40meV
$\mathcal{U}_2$	57meV	58meV
V_{dis}	10meV	10meV
C	4.11meV V^{-1}	7.8meV V^{-1}
V_{th_1}	0V	3.8V
V_{th_2}	0V	2.56V

TABLE S3. Numerical values of the model parameters used in the simulations for the InSb nanoflag. The subscripts $j = 1, 2$ indicate the normal region of the two junctions, respectively.

sits at $y = W_1 + d$. These different offsets enter as different values of y_0 in Eq. (11) for the two junctions. Under these assumptions, the area of the SQUID pierced by the magnetic flux is defined as $A_{\mathrm{eff}}^{\mathrm{th}} = L\left(d + \frac{W_1+W_2}{2}\right)$. In the simulations, we fix d such that $A_{\mathrm{eff}}^{\mathrm{th}}$ matches the A_{eff} extracted from the experimental data on the SQUID pattern, so as to correctly reproduce the SQUID periodicity.

Concerning the chemical potential appearing in the theoretical model, this is assumed to be tuned by the back-gate potential according to the following phenomenological relation

$$\mu = C(V_{\mathrm{bg}} - V_{\mathrm{th}}), \tag{15}$$

with the threshold voltage which can be different for the two normal regions and is set to zero in the superconducting leads.

Table S3 lists the parameters used for the simulation of the symmetric and asymmetric SQUID. Although the parameters have been fine-tuned to better fit the experimental data, it should be underlined that they all possess the correct order of magnitude and that small variations of the reported values do not significantly alter the correspondence with the experimental data.

S7. ADDITIONAL DATA

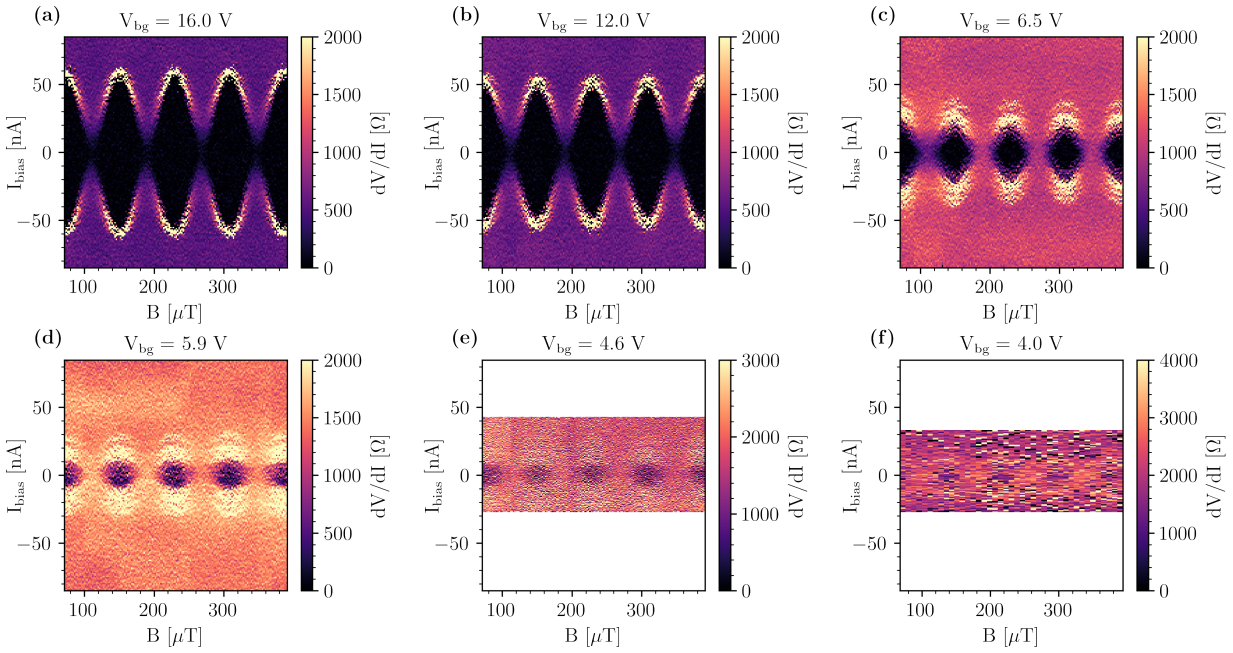

FIG. S6. Symmetric SQUID interference patterns for different back-gate voltages.

Supplementary interference patterns for different back-gate voltages for the symmetric SQUID are shown in Fig. S6. No loss of interference is observed in lowering the back-gate voltage, meaning that in both arms the supercurrent pinches off at the same V_{bg}, which is $\sim 4.0\,\mathrm{V}$, consistent with the symmetric design and with the single-threshold picture of the normal-state characterisation.

The asymmetric SQUID patterns for various V_{bg} values are shown in Fig. S7. An asymmetry in the critical currents, $I_{c1} \neq I_{c2}$, is observed across all the explored V_{bg} range. At approximately $V_{\mathrm{bg}} \sim 4.0\,\mathrm{V}$, one of the junctions pinches off and does not support supercurrent transport, leading to the absence of observable SQUID interference. As the chemical potential is brought by the back-gate into the band-gap of the semiconductor, the supercurrent transport of the Josephson junction transits from a SNS-junction regime to an exponentially suppressed tunneling regime, where $I_{\mathrm{c}} \sim 0\,\mathrm{nA}$ given the electrode spacing of $L \approx 200\,\mathrm{nm}$.

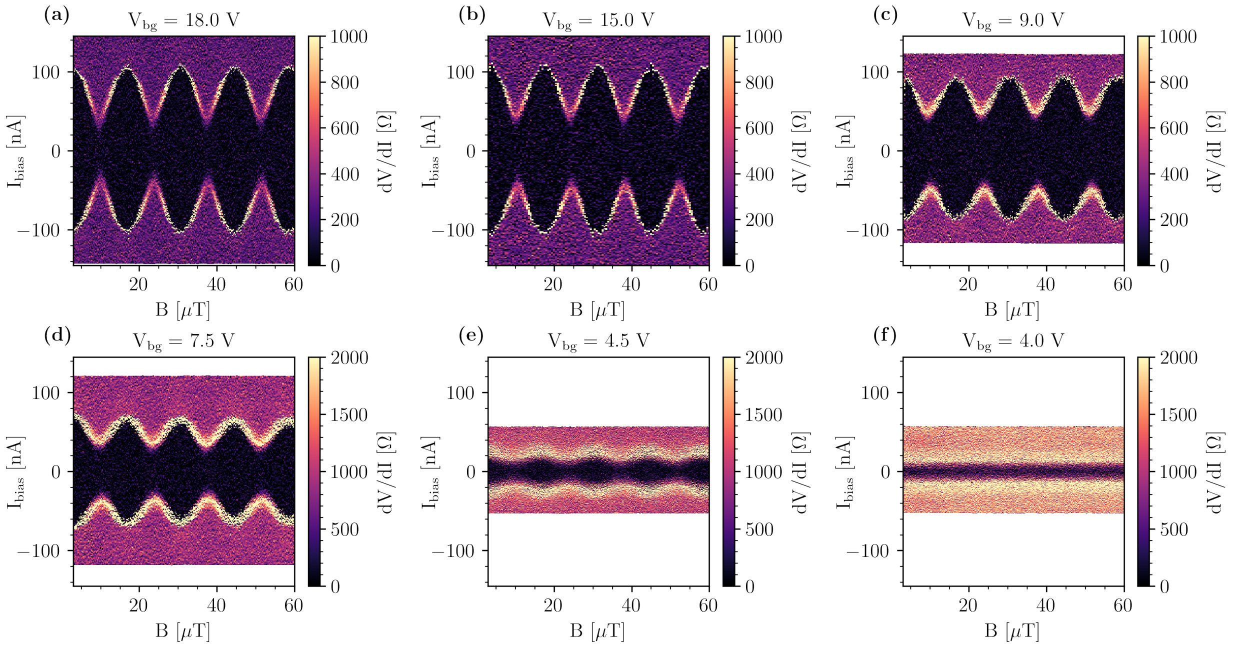

FIG. S7. Asymmetric SQUID interference patterns for different back-gate voltages.

S8. JOSEPHSON DIODE EFFECT IN THE ASYMMETRIC SQUID

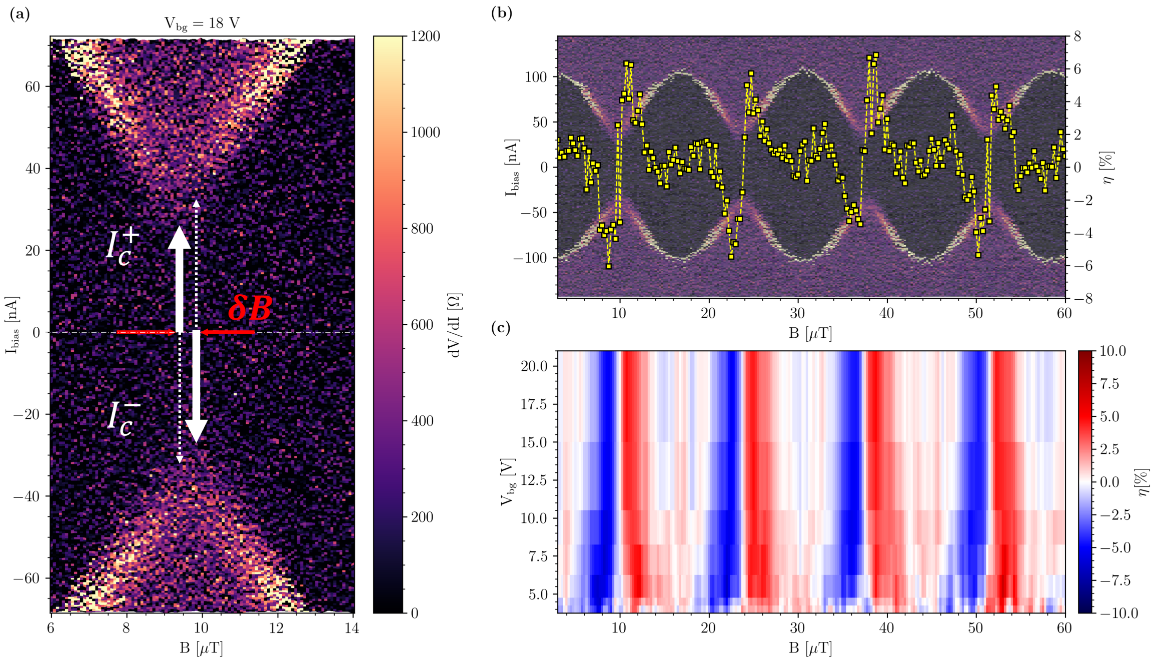

FIG. S8. (a) Zoom near the minima in the interference pattern of the asymmetric SQUID. $T = 350\,\mathrm{mK}, V_{bg} = 18\,\mathrm{V}$. (b) The rectification coefficient η (yellow squares) superimposed on the interference pattern, highlighting its periodicity with the applied magnetic field. (c) Colour-map of η against the back-gate voltage.

Starting from a high value ($I > I_c^+$), we sweep down to $I < I_c^-$ and then perform the reverse sweep. This allows us to record both the switching and the retrapping currents in forward and backward sweep direction (I_{sw}^+, I_{rt}^+, I_{sw}^-, I_{rt}^-). As stated in the main text, I_{sw}^+ and I_{rt}^+ have the same value, and thus we call them I_c^+ (same for $I_c^- \equiv I_{sw}^- = I_{rt}^-$). In the resulting SQUID interference patterns, it is found that the minima in magnetic field of the two quantities I_c^+ and I_c^- are slightly shifted, indicating the presence of Josephson diode effect (JDE). Fig. S8(a) shows a zoom around the minima in the interference pattern presented in Fig. 4(a) of the main text. In this case, the minima are shifted by $\delta B \sim 1\,\mu\mathrm{T}$, corresponding to $\sim 0.07\ \Phi_0$. To quantify the amount of diode effect, it is common to use the rectification coefficient η:

$$\eta = \frac{I_{c+} - |I_{c-}|}{I_{c+} + |I_{c-}|} \tag{16}$$

This is plotted against the magnetic field in Fig. S8(b), where for clarity the corresponding interference pattern is represented in the background. To improve the signal-to-noise ratio, averaging between switching and retrapping current is performed, since no notable difference is present between those two quantities, and JDE is observed in both retrapping and switching currents. An oscillatory behaviour in η with the same periodicity as the SQUID pattern is present, and η can be tuned from $\sim 6\%$ to $\sim -6\%$. The magnetic fields for which η changes sign are those for which the critical current is almost at its maximum and minimum value. This is particularly evident in Fig. S8(c), where a colour-map illustrates both the magnetic field dynamics and the gate-tunability of η. The rectification coefficient goes to zero for $V_{\mathrm{bg}} = 4\,\mathrm{V}$. The fact that the back-gate does not reverse the sign of η indicates that the Josephson junction with the higher critical current is the same for every V_{bg} explored. In fact, in SQUIDs where the two I_{ci} vs V_{bg} cross (Ref. [11]) the JDE pattern reports a sign-reversal at the back-gate value for which inversion symmetry is restored. Our observations are consistent with theoretical predictions expected for SQUIDs: three conditions should be present at the same time for a SQUID to display JDE [12]:

1. The external flux must not be equal to a an integer multiple of half the flux quantum: $\Phi \neq n\frac{\Phi_0}{2}$;

2. The transmission of the Josephson junctions (transparency of the interface) must not be equal;

3. At least one Josephson junction needs to be highly transmitting to have a sizable higher harmonic content in the CPR.

Our results thus provide support that InSb nanoflag-based Josephson junctions are highly transmissive and present higher harmonic content in the CPR.

S9. FRAUNHOFER-LIKE INTERFERENCE

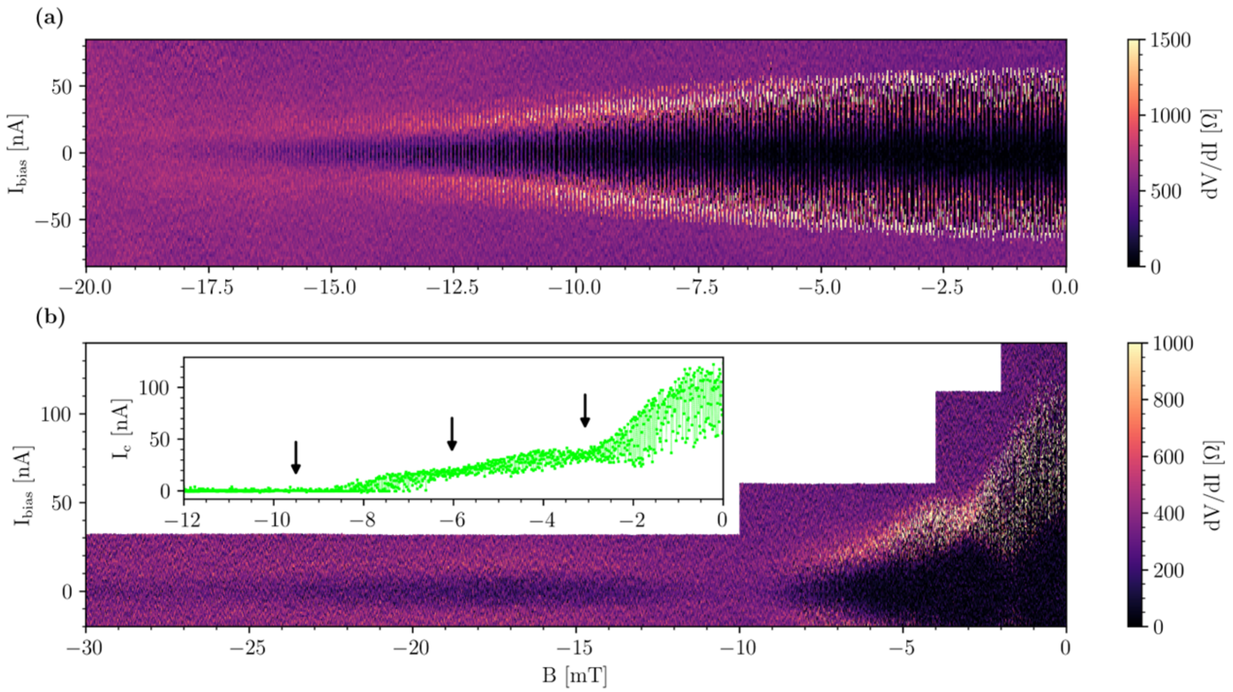

FIG. S9. Fraunhofer patterns of the SQUIDs. (a) Symmetric device, $V_{bg} = 20\,V$. (b) Asymmetric device, $V_{bg} = 15\,V$. Inset: Critical current (I_c) as a function of magnetic field. SQUID modulation of I_c is locally suppressed at the magnetic field values indicated by black arrows at $B = -3.0 \pm 0.5\,mT$, $B = -6.0 \pm 0.5\,mT$, and $B = -9.5 \pm 1\,mT$. $T = 350\,mK$.

When the magnetic flux over one or both individual junctions, $\Phi_{JJ,i}$, becomes of the same order of magnitude as the superconducting flux quantum, the effects of the SQUID and the Fraunhofer-like interference of the single junctions superimpose, and a low-frequency Fraunhofer envelope modulates the high-frequency SQUID interference pattern. Figure S9 explores the Fraunhofer-like regime of SQUIDs for both geometries, at high back-gate voltage (20 V and 15 V for the symmetric and asymmetric case, respectively). The two individual junctions of the symmetric device modulate the SQUID oscillations with a Gaussian envelope, as shown in Fig. S9(a). For InSb nanoflags, this kind of supercurrent modulation has already been reported for junctions in the narrow configuration [13–15]. In previous works, the absence of pronounced side lobes was attributed to a limited W/L ratio of the junctions [15–18]. For the symmetric SQUID, both junctions have $W/L \sim 2$, slightly lower than the previous devices, further supporting this picture. From Fig. S9(a), it is found

that at a magnetic field of $B_0 = 18.5\,\mathrm{mT}$ the SQUID interference is no more visible. Along the Fraunhofer pattern, no beating of the modulation amplitude is visible. Hence, both junctions have the same area, consistent with the symmetric geometry. The effective area extracted from this measurement is $0.11\,\mu\mathrm{m}^2$, in agreement with the geometrical area of $(L + 2\lambda_\mathrm{L}) \times W = 0.11\,\mu\mathrm{m}^2$ measured from SEM images (with λ_L the London penetration depth).

The same measurement for the asymmetric SQUID is displayed in Fig. S9(b). In this case, the Josephson junctions have different areas ($A_{\mathrm{JJ},1}/A_{\mathrm{JJ},2} \approx 3.1$), resulting in a visible beating in the Fraunhofer envelope modulating the SQUID oscillations. The two different configurations of the junctions result in $W/L = 2.5$ for the narrow junction and $W/L = 8.5$ for the wide one. The inset of Fig. S9(b) shows that the SQUID modulation amplitude is suppressed for magnetic fields of $B = -3.0 \pm 0.5\,\mathrm{mT}$, $B = -6.0 \pm 0.5\,\mathrm{mT}$, and $B = -9.5 \pm 1\,\mathrm{mT}$. For the first two field values, the total supercurrent is not zero, suggesting that for these field values only one junction carries a negligible supercurrent because it is at a Fraunhofer minimum. Instead, total suppression of the supercurrent of the whole SQUID occurs for $B = -9.5\,\mathrm{mT}$. These observations suggest that the resonant features at the smaller field values can be attributed to the larger junction, while the feature at $-9.5\,\mathrm{mT}$ is attributed to a Fraunhofer minimum of both junctions. This is consistent with the fact that the ratio $A_{\mathrm{JJ},1}/A_{\mathrm{JJ},2}$ has almost an integer value and that the first minimum of the smaller junction corresponds approximately to the third minimum of the larger junction. Converting these magnetic fields to areas leads to $A_\mathrm{eff} = 1.55 \cdot A_\mathrm{geo}$ for both junctions, which we attribute to the flux-focusing effect.

S10. MAGNETOMETER RESPONSE FOR THE SYMMETRIC SQUID

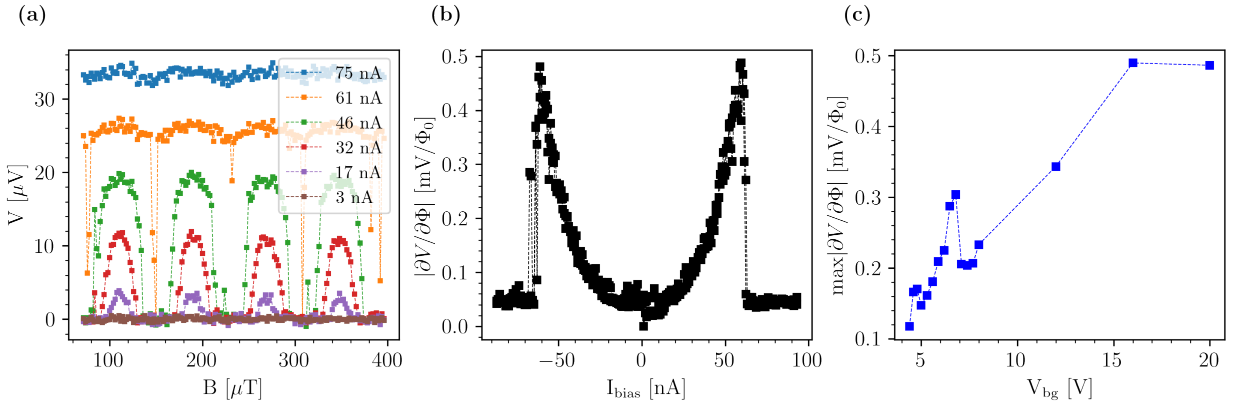

FIG. S10. (a) $V-B$ characteristics for different values of current bias. $T = 350\,\text{mK}, V_{\text{bg}} = 20\,\text{V}$. (b) Corresponding voltage responsivity as a function of current bias. (c) Maximum responsivity as a function of back-gate voltage.

In the main text, the dissipative response of the asymmetric device was presented. For comparison, in this section we report details regarding the dissipative response of the symmetric SQUID. Fig. S10(a) shows the $V-B$ curves of the device at $V_{\text{bg}} = 20\,\text{V}$, for different values of the current bias. Tuning I_{bias} to $\sim 50\,\text{nA}$ allows to reach a voltage drop modulation of $\Delta V \sim 20\,\mu\text{V}$, similar to the asymmetric device. This corresponds to an optimal voltage transfer function $V_\Phi = 0.5\,\text{mV}/\Phi_0$ at $V_{\text{bg}} = 20\,\text{V}$, (panel (b)), which is gate-tunable and presents its maximum value for V_{bg} above $15\,\text{V}$ (panel (c)).

Using the value of the preamplifier input voltage noise, 4 $\text{nV}/\sqrt{\text{Hz}}$, we find that the magnetic flux noise at low frequency is $S_\Phi^{1/2} = 8.0 \times 10^{-6}\ \Phi_0/\sqrt{\text{Hz}}$ at $T = 350\,\text{mK}$.

---

[1] Verma, I., Salimian, S., Zannier, V., Heun, S., Rossi, F., Ercolani, D., Beltram, F., and Sorba, L., High-Mobility Free-Standing InSb Nanoflags Grown on InP Nanowire Stems for Quantum Devices. *ACS Appl. Nano Mater.* **4**, (2021), 5825–5833.

[2] Suyatin, D. B., Thelander, C., Björk, M. T., Maximov, I., and Samuelson, L., Sulfur passivation for ohmic contact formation to InAs nanowires. *Nanotechnology* **18**, (2007), 105307.

[3] Gubin, A. I., Il'in, K. S., Vitusevich, S. A., Siegel, M., and Klein, N., Dependence of magnetic

penetration depth on the thickness of superconducting Nb thin films. *Phys. Rev. B* **72**, (2005), 064503.

[4] Clayton, R. P. (2009) *Inductance: Loop and Partial*. Wiley-IEEE Press.

[5] Granata, C. and Vettoliere, A., Nano Superconducting Quantum Interference device: A powerful tool for nanoscale investigations. *Phys. Rep.* **614**, (2016), 1–69.

[6] Annunziata, A. J., Santavicca, D. F., Frunzio, L., Catelani, G., Rooks, M. J., Frydman, A., and Prober, D. E., Tunable superconducting nanoinductors. *Nanotechnology* **21**, (2010), 445202.

[7] Blonder, G. E., Tinkham, M., and Klapwijk, T. M., Transition from metallic to tunneling regimes in superconducting microconstrictions: Excess current, charge imbalance, and supercurrent conversion. *Phys. Rev. B* **25**, (1982), 4515–4532.

[8] Furusaki, A., DC Josephson effect in dirty SNS junctions: Numerical study. *Physica B* **203**, (1994), 214–218.

[9] Asano, Y., Numerical method for dc Josephson current between d-wave superconductors. *Phys. Rev. B* **63**, (2001), 052512.

[10] Sancho, M. P. L., Sancho, J. M. L., Sancho, J. M. L., and Rubio, J., Highly convergent schemes for the calculation of bulk and surface Green functions. *J. Phys. F: Met. Phys.* **15**, (1985), 851.

[11] Wu, X., Wang, J.-Y., Su, H., Yan, S., Pan, D., Zhao, J., Zhang, P., and Xu, H. Q., Tunable superconducting diode effect in higher-harmonic InSb nanosheet interferometers. *New J. Phys.* **27**, (2025), 023031.

[12] Souto, R. S., Leijnse, M., and Schrade, C., Josephson Diode Effect in Supercurrent Interferometers. *Phys. Rev. Lett.* **129**, (2022), 267702.

[13] Salimian, S., Carrega, M., Verma, I., Zannier, V., Nowak, M. P., Beltram, F., Sorba, L., and Heun, S., Gate-controlled supercurrent in ballistic InSb nanoflag Josephson junctions. *Appl. Phys. Lett.* **119**, (2021), 214004.

[14] Iorio, A., et al., Half-integer Shapiro steps in highly transmissive InSb nanoflag Josephson junctions. *Phys. Rev. Res.* **5**, (2023), 033015.

[15] Turini, B., Salimian, S., Carrega, M., Iorio, A., Strambini, E., Giazotto, F., Zannier, V., Sorba, L., and Heun, S., Josephson Diode Effect in High-Mobility InSb Nanoflags. *Nano Lett.* **22**, (2022), 8502–8508.

[16] Barzykin, V. and Zagoskin, A. M., Coherent transport and nonlocality in mesoscopic SNS junctions: anomalous magnetic interference patterns. *Superlattice. Microst.* **25**, (1999), 797–807.

[17] Cuevas, J. C. and Bergeret, F. S., Magnetic Interference Patterns and Vortices in Diffusive SNS Junctions. *Phys. Rev. Lett.* **99**, (2007), 217002.

[18] Angers, L., Chiodi, F., Montambaux, G., Ferrier, M., Guéron, S., Bouchiat, H., and Cuevas, J. C., Proximity dc squids in the long-junction limit. *Phys. Rev. B* **77**, (2008), 165408.
